# Genome-Wide Identification and Expression Profiling of Cytokinin Oxidase/Dehydrogenase (*CKX*) Genes Reveal Likely Roles in Pod Development and Stress Responses in Oilseed Rape (*Brassica napus* L.)

**DOI:** 10.3390/genes9030168

**Published:** 2018-03-16

**Authors:** Pu Liu, Chao Zhang, Jin-Qi Ma, Li-Yuan Zhang, Bo Yang, Xin-Yu Tang, Ling Huang, Xin-Tong Zhou, Kun Lu, Jia-Na Li

**Affiliations:** 1 Chongqing Rapeseed Engineering Research Center, College of Agronomy and Biotechnology, Southwest University, Chongqing, 400715, China; liupu28@163.com (P.L.); 18083606406@163.com (C.Z.); jinqima1996@163.com (J.Q.M.); liyuanzhang0215@163.com (L.Y.Z.); sheepneck@hotmail.com (B.Y.); ttxy1996@163.com (X.Y.T.); lingheather@163.com (L.H.); wjzxt22@163.com (X.T.Z.); drlukun@swu.edu.cn (K.L.); 2 Academy of Agricultural Sciences, Southwest University, Chongqing, 400715, China

**Keywords:** *Brassica napus*, cytokinin oxidase/dehydrogenases (CKX), expression analysis, differentially expressed genes, silique length, harvest index.

## Abstract

Cytokinin oxidase/dehydrogenases (CKXs) play a critical role in the irreversible degradation of cytokinins, thereby regulating plant growth and development. *Brassica napus* is one of the most widely cultivated oilseed crops worldwide. With the completion of whole-genome sequencing of *B. napus*, genome-wide identification and expression analysis of the *BnCKX* gene family has become technically feasible. In this study, we identified 23 *BnCKX* genes and analyzed their phylogenetic relationships, gene structures, conserved motifs, protein subcellular localizations, and other properties. We also analyzed the expression of the 23 *BnCKX* genes in the *B. napus* cultivar Zhong Shuang 11 (‘ZS11’) by quantitative reverse-transcription polymerase chain reaction (qRT-PCR), revealing their diverse expression patterns. We selected four *BnCKX* genes based on the results of RNA-sequencing and qRT-PCR and compared their expression in cultivated varieties with extremely long versus short siliques. The expression levels of *BnCKX5-1*, *5-2*, *6-1*, and *7-1* significantly differed between the two lines and changed during pod development, suggesting they might play roles in determining silique length and in pod development. Finally, we investigated the effects of treatment with the synthetic cytokinin 6-benzylaminopurine (6-BA) and the auxin indole-3-acetic acid (IAA) on the expression of the four selected *BnCKX* genes. Our results suggest that regulating *BnCKX* expression is a promising way to enhance the harvest index and stress resistance in plants.

## 1. Introduction

Cytokinins promote cell division, regulate root and shoot differentiation synergistically with auxin [1,2,3,4,5], release apical dominance, and delay leaf senescence [6,7,8]. Cytokinins also function in responses to various biotic and abiotic stresses [9,10]. Most naturally occurring cytokinins are adenine derivatives whose N^6^-side chain is substituted by an isoprenoid or aromatic side-chain. Cytokinin biosynthetic and metabolic enzymes play critical roles in maintaining cytokinin concentrations in individual cells or tissues at the proper level for normal functioning [11]. Isopentenyl transferases (IPT) catalyze a step in cytokinin biosynthesis, and cytokinin oxidase/dehydrogenases (CKXs) catalyze the irreversible degradation of cytokinin [12,13,14]. CKXs act on specific substrates such as isopentenyladenine and isopentenyladenine riboside [15]. These enzymes catalyze the irreversible degradation of cytokinins and their derivatives by removing their N^6^-substituted isoprene chains [16]. In this catalytic reaction, CKX enzymes display a dual catalytic mode in conjunction with molecular oxygen or other specific substances, which act as electron acceptors [17]. CKXs exhibit a relatively high degree of thermal stability, and the optimal pH for their activity depends on their natural substrates [18]. CKXs can function as flavin enzymes by covalently binding to flavin adenine dinucleotide (FAD) via a histidine residue, and they contain both FAD- and CK-binding domains [19]. Most CKX proteins undergo post-translational glycosylation, which might influence their molecular weights and localization [15].

CKX proteins are encoded by a small gene family [20]. *CKX* family genes and some putative members have been identified or functionally expressed in heterologous hosts, including various plant species such as *Arabidopsis thaliana* [5], *Oryza sativa* [21], *Nicotiana tabacum* [22], *Zea mays* [23,24], *Triticum aestivum* [25,26], *Hordeum vulgare* [25,26], *Brassica rapa* [27], *Gossypium hirsutum* [28], *Setaria italica* [29], *Fragaria vesca* [30], *Dendrobium* orchid [31], *Pisum sativum* [32] and *Glycine max* [33]. *A. thaliana* contains seven *CKX* genes (*AtCKX1*–*AtCKX7*) [5] with differing biochemical characteristics, subcellular localizations and expression patterns. Detailed analysis of transgenic *Arabidopsis* plants expressing each of these genes revealed that all of the transformants exhibited reduced cytokinin levels and developmental changes in roots and shoots, suggesting that the spatial and temporal expression patterns of *CKX*s might be related to the diverse functions of cytokinins [5,34]. Furthermore, overexpressing *AtCKX1* and *AtCKX2* in transgenic *Centaurium erythraea* led to decreased morphogenetic potential but had no effect on biomass production compared to the untransformed control [35]. The reduced expression of *OsCKX2* in rice greatly increased cytokinin accumulation in the inflorescence meristem and increased the number of reproductive organs [21]. Similarly, silencing of *HvCKX1* in barley led to lower CKX activity, thereby increasing seed yields and root weight [36].

Oilseed rape (*Brassica napus* L.), one of the most widely cultivated oilseed crops worldwide, was derived from natural hybridization between *B. rapa* and *Brassica oleracea*. A major goal in breeding oilseed rape is to enhance its yield and harvest index. The number of siliques per plant, number of seeds per pod and thousand-seed weight affect yield in this crop, and there is a significant positive correlation between silique length and seed weight [37,38]. The silique pericarp functions as a source organ to provide photosynthates to the developing seed; therefore, silique length may affect seed filling [39]. 

Numerous studies have shown that *CKX* genes strongly contribute to yield and stress responses [40]. In the current study, to identify genes that might help determine silique length and function in pod development in *B. napus,* we identified 23 *CKX* genes in *B. napus* and constructed a phylogenetic tree to evaluate the phylogenetic relationships between BnCKX proteins and those of other species. We also analyzed the properties and expression patterns of *BnCKXs* and identified differentially expressed genes (DEGs) between plants with long versus short siliques. Finally, we investigated the responses of selected *BnCKX* genes to hormone treatment. 

## 2. Materials and Methods

### 2.1 Identification of CKXs in B. napus, B. rapa, and B. oleracea

The nucleotide and protein sequences of the *AtCKXs* were acquired from The *Arabidopsis* Information Resource database (https://www.arabidopsis.org/index.jsp). *BnCKXs*, *BrCKXs* and *BoCKXs* were identified through BLASTP analysis [41] of *AtCKXs* against the *Brassica* database (BRAD, http://brassicadb.org/brad/index.php) [42]. The CKX enzymes contain both a FAD-binding and a cytokinin-binding domain. Therefore, InterProScan (http://www.ebi.ac.uk/interpro/) was used to exclude any genes that did not contain these domains [43]. 

### 2.2. Multiple Sequence Alignment and Phylogenetic Analysis

Multiple sequence alignment of the protein sequences from the four species mentioned above was performed with MEGA7.0 [44] using default parameters. The conserved blocks of all predicted sequences were identified using the Gblocks program [45], and substitution saturation tests were performed with DAMBE program. To increase the accuracy of the predicted phylogenetic relationships of CKX family members of the four species, phylogenetic trees were constructed using three methods. A Neighbor-Joining tree (NJ tree) was generated via bootstrap analysis with 1000 replicates using MEGA7.0. A Maximum Likelihood tree (ML tree) was generated using the online program ATGC phyML with default parameters (http://www.atgc-montpellier.fr/phyml/) [46]. A Bayesian Inference tree (BI tree) was generated using the MrBayes program and ModelGenerator software [47]. Finally, the optimum topology was chosen using Tree-puzzle-5.3 and CONSEL software [48]. 

### 2.3. Chromosomal Locations, Gene Structures and Protein Profiles of *BnCKXs*

Chromosomal position information for the *BnCKXs* was obtained from the *B. napus* Genome Browser, and the genes were mapped onto chromosomal linkage groups with MapChart [49]. To investigate the exon/intron organization of the *BnCKXs,* the gene structures were analyzed using the Gene Structure Display Server (http://gsds.cbi.pku.edu.cn/) [50]. Protein motifs were identified using MEME (http://meme-suite.org/tools/meme) [51]: the maximum number of motifs was set to 20 and default settings were used for other parameters. Annotations of the identified motifs were obtained from InterProScan. The isoelectric points (pI) and molecular weights (Mw) of the BnCKXs were calculated using the ExPASy proteomics server database (http://www.expasy.org/tools/) [52]. The subcellular localizations of the BnCKX proteins were determined using WoLF PSORT (http://www.genscript.com/tools/wolf-psort) [53].

### 2.4. Sequence Analysis of the *BnCKXs* Promoters

The promoter sequences (1,500 bp upstream of the start codon) of the *BnCKX*s were retrieved from the *B. napus* genome database (http://www.genoscope.cns.fr/brassicanapus/) [54]. PlantCARE (http://bioinformatics.psb.ugent.be/webtools/plantcare/html/) [55] was used to identify putative *cis*-regulatory elements in the *BnCKX* promoters.

### 2.5. Plant Materials, Growth Conditions, and Treatments

The plants were cultivated at an experimental farm in Beibei, Chongqing, China (29°45′ N, 106°22′ E), for two consecutive years (2016 and 2017). To analyze the expression patterns of the *BnCKXs*, different tissues were sampled, including mature leaves (ML), buds (B), flowers (F), roots (R), stems (S), and stalks (ST) of *B. napus* cultivar Zhong Shuang 11 (‘ZS11’) during the full-bloom stage. After the termination of flowering, silique pericarps and seeds were collected at different time points (0, 7, 14, 21, 30, and 40 days after flowering, DAF). The silique pericarps and seeds (0, 7, 14, 21, 30, and 40 DAF) were collected from plants with extreme siliques lengths from Zhong Shuang 4 (‘ZS4’) and Ning You 12 (‘NY12’) *B. napus* cultivar materials. At several stages of pod development, 10 pods in the middle of the main stem of the inflorescence were taken from three individual plants to measure the length of the siliques, and the mean values were obtained (Table S1). For hormone treatments, four-week-old oilseed rape seedlings were treated with indole-3-acetic acid (IAA, 10 µM) or 6-benzylaminopurine (6-BA, 10 µM). The plant growth conditions were described in Lu et al. [56]. Leaves were harvested at 0, 1, 3, 6, 12 and 24 h after IAA treatment and at 0, 1, 3, 6, 12, 24 and 48 h after 6-BA treatment. The samples were rapidly frozen in liquid nitrogen and stored at -80°C until use.

### 2.6. RNA-seq Analysis

To identify DEGs and to analyze the tissue-specific expression of the *BnCKXs*, publically available *B. napus* RNA-sequencing (RNA-Seq) data were downloaded and used to construct a heatmap with R-Studio. Data for the 23 *BnCKX* genes were extracted from the RNA-Seq database (accession number SRP072900), including expression data from eight different tissues collected from four *B. napus* materials with an extremely high or low harvest index grown in two different environments (Chongqing and Yunnan province, China) [57].

### 2.7. RNA Isolation and qRT-PCR Analysis

Total RNA was extracted with an RNeasy Extraction kit (Invitrogen, Carlsbad, CA, USA). First-strand cDNA was synthesized using M-MLV transcriptase (TaKaRa Biotechnology, Dalian, China) after DNase I treatment according to the manufacturer’s instructions. Quantitative reverse-transcription polymerase chain reaction (qRT-PCR) was then conducted to measure the expression levels of the 23 *BnCKXs* in various tissues of ZS11. After analyzing the RNA-Seq data and the expression patterns of all *BnCKXs* in ZS11, qRT-PCR was also conducted to measure the expression of selected genes (*BnCKX5-1*, *BnCKX 5-2*, *BnCKX 6-1* and *BnCKX7-1*) in seeds and silique pericarps at different stages in *B. napus* plants with extremely long versus short siliques. The qRT-PCR was performed as described by Ma [58]. Gene-specific primer pairs for the 23 *BnCKXs* were designed using Primer Premier 6.0 (Table S2) [59]. Melting curve analysis and agarose gel electrophoresis were performed to confirm the specificity of the product. Three biological replicates with three technical replicates were performed for each reaction. The *BnActin7* gene served as an endogenous reference gene. The calculation of the gene relative expression levels followed the 2^−ΔΔCT^ method described by Livik and Schmittgen. [60]

## 3. Results

### 3.1. Identification and Phylogenetic Analysis of *BnCKXs*

**Figure 1:**
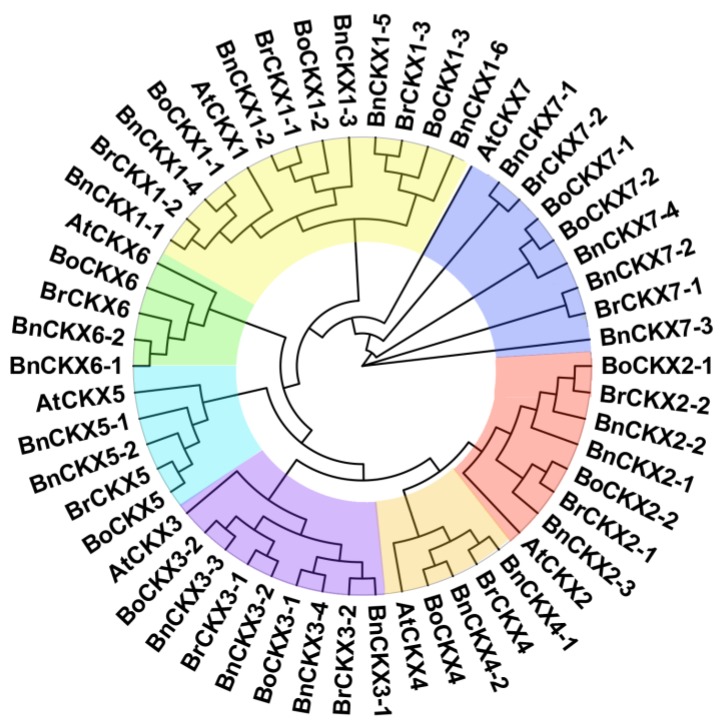
Bayesian Inference tree of 54 CKX protein sequences from *Arabidopsis thaliana, Brassica rapa, Brassica oleracea*, and *Brassica napus.*

Using *A. thaliana* AtCKX protein sequences as queries, seven *AtCKXs*, 23 *BnCKXs*, 12 *BoCKXs* and 12 *BrCKXs* were identified via BLASTP analysis. Among the three phylogenetic trees, the BI tree had better topology than NJ, and ML trees and was therefore used to analyze the evolutionary relationships of CKX proteins. The 54 CKX proteins were grouped into seven distinct clades, with AtCKX1-7 and the corresponding *B. rapa*, *B. oleracea*, and *B. napus* subfamily members in each clade, hereafter referred to as groups I-VII, respectively. (Figure 1). Basic information about the *BnCKXs* is shown in Table 1. The encoded proteins vary from 336 (BnCKX2-2) to 768 (BnCKX2-1) amino acid (aa) residues in length, and the corresponding molecular weights of the BnCKXs range from 38.17 kDa (BnCKX2-2) to 87.02 kDa (BnCKX2-1). The BnCKXs also exhibit a wide range of pI values from 4.92 (BnCKX7-2) to 9.2 (BnCKX1-4). The pI values of proteins in groups I, II, VI and VII (except BnCKX7-3, pI value is 7.93) are less than 6.0, while those in group III and IV are greater than 7.0.

### 3.2. Chromosomal Localization

**Figure 2:**
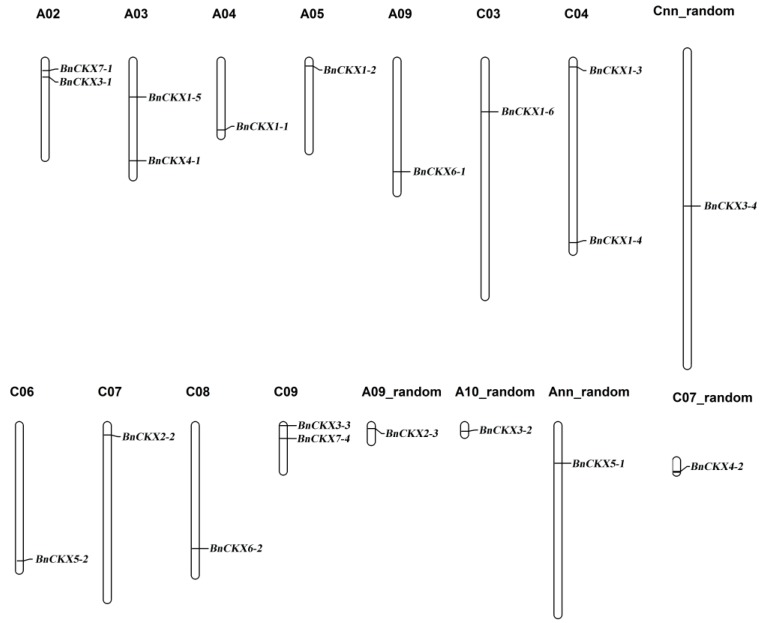
Distribution of *BnCKX* genes in the *B. napus* genome. The chromosomal position of each *BnCKX* gene was mapped to the *B. napus* genome: the chromosome number is indicated above each chromosome. The scale is in megabases (Mb). Ann and Cnn are pseudo-molecule chromosomes. Random: The specific position of gene is still unknown.

Chromosome mapping revealed that the *BnCKXs* are unevenly distributed throughout the chromosomes. Chromosome A02, A03, A10, C04 and C09 each contain two *BnCKX* genes, whereas chromosomes A04, A05, A09, C03, C06, C07, C08, A07-random, A09-random, A10-random, Ann-random, C07-random and Cnn-random contain only one. *BnCKX1-3* and *BnCKX1-4* (Group III) are located on chromosome C04, while *BnCKX7-2* and *BnCKX7-3* (Group VI) are located on chromosome A10 (Figure 2). 

### 3.3. Gene Structures and Protein Profiles of *BnCKXs*

**Figure 3:**
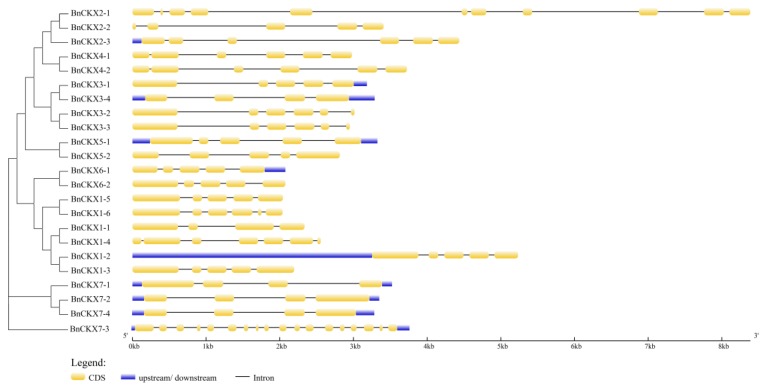
Exon–intron structures of the *BnCKX* genes according to their phylogenetic relationships. An unrooted phylogenetic tree was constructed with 1000 bootstrap replicates based on the full-length sequences of BnCKX proteins. Exon–intron structure analysis of the *BnCKX* genes was performed using the online tool Gene Structure Display Server (GSDS). The lengths of the exons and introns of each *BnCKX* gene are drawn to scale.

**Figure 4:**
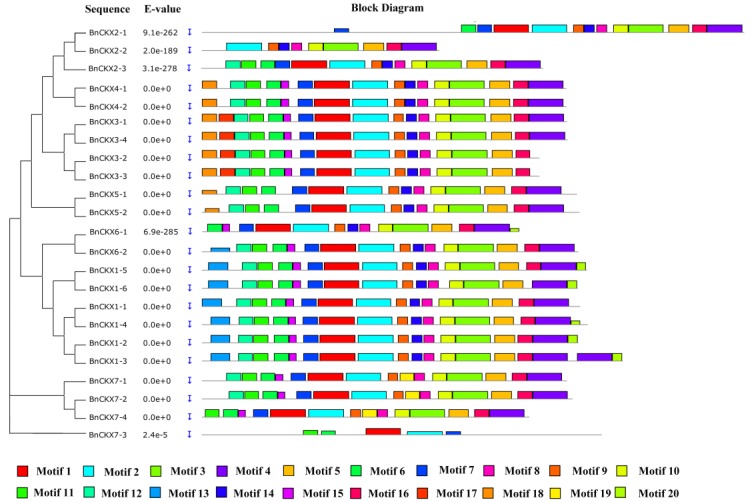
The conserved motifs of the BnCKX proteins according to their phylogenetic relationships. The conserved motifs of the BnCKX proteins were identified by MEME. Gray lines represent non-conserved sequences, and each motif is indicated by a colored box (numbered at the bottom). The lengths of the motifs in each protein are drawn to scale.

Figure 3 shows the gene structures of the *BnCKXs*. Overall, the closer the relationship between genes, the more similar their structures. Among the *BnCKXs*, the gene pairs *BnCKX1-2* and *BnCKX1-3*, *BnCKX4-1* and *BnCKX4-2*, *BnCKX7-2* and *BnCKX7-4*, and *BnCKX3-2* and *BnCKX3-3* showed the highest similarity in terms of exon/intron number, distribution and length. However, *BnCKX7-3* and *BnCKX2-1* are strikingly different from other members of the same group in terms of exon numbers. *BnCKX7-3* and *BnCKX2-1* contained 18 and 11 exons, respectively. The MEME tool identified 20 conserved motifs in the BnCKXs (Figure 4). Motif 2 is present in all family members, whereas motifs 1, 6, and 7 are present in all family members except BnCKX2-2. Motifs 3, 4, 5, 8, 9 and 10 are present in all family members except BnCKX2-2. Motifs 13 and 20 are present only in BnCKX5-3 and BnCKX5-4, and motif 17 is found only in BnCKX5-7. Overall, except for BnCKX2-1, BnCKX2-2, and BnCKX7-3, the BnCKX proteins contain highly similar motifs in terms of type, order and number. We annotated the conserved motifs using the InterProScan program. Motifs 2 and 3 are associated with cytokinin dehydrogenase and the vanillyl-alcohol oxidase domain, whereas motifs 1, 2, 4, and 6 are annotated as FAD/cytokinin binding domains that can bind both FAD and cytokinins as substrates. The other motifs could not be annotated. 

Subcellular localization analysis showed that seven of the 23 BnCKX proteins were predicted to be located in the vacuole, six in the mitochondria, five in the chloroplast, two in the nucleus, and two in the cytosol. The remaining proteins might be excreted into the extracellular space. BnCKX1-5 and 1-6 might be located in the mitochondria, and the BnCKX3s are localized to the vacuole. 

### 3.4. Cis-Acting Elements in the *BnCKXs*

*Cis*-acting elements in gene promoters, which serve as the binding sites of transcription factors, are important for regulating the initiation of gene transcription and for transcription efficiency. We identified 82 types of *cis*-acting elements in the promoters of the *BnCKXs* (Table S3). TATA-box and CAAT-box are core promoter elements, while light-responsive elements are the most varied *cis*-acting elements (25 types) and are present at the highest frequency (168 instances). Hormone-responsive *cis*-acting elements also account for a large proportion of elements, including auxin-responsive elements (TGA-element, AuxRR-core and TGA-box), MeJA-responsive elements (CGTCA-motif and TGACG-motif), gibberellin-responsive elements (GARE-motif, P-box and TATC-box), ethylene-responsive elements (ERE), salicylic acid-responsive elements (TCA-element) and abscisic acid-responsive elements (ABRE). 

We also identified some environmental stress-responsive element in these genes. For instance, we identified: box-W1 and box E, which are fungal elicitor-responsive elements; ELI-box3, an elicitor-responsive element; WUN-motif, a wounding-responsive element; box S, a wounding- and pathogen-responsive element; LTR, involved in low-temperature responses; HSE, which functions in heat stress responses, MBS, a drought-inducible element and TC-rich repeats, which function in defense and stress responses. 

Notably, some *cis*-acting elements in *BnCKX* might function in tissue-specific expression, such as the following: CAT-box and CCGTCC-box, which function in meristem-specific activation; Skn-1_motif and GCN4_motif, which are involved in endosperm expression; RY-element, involved in seed-specific regulation; as 1, involved in root-specific expression and HD-Zip 1 and HD-Zip 2 control palisade mesophyll cell differentiation and leaf morphology, respectively.

### 3.5. RNA-Seq Analysis

**Figure 5:**
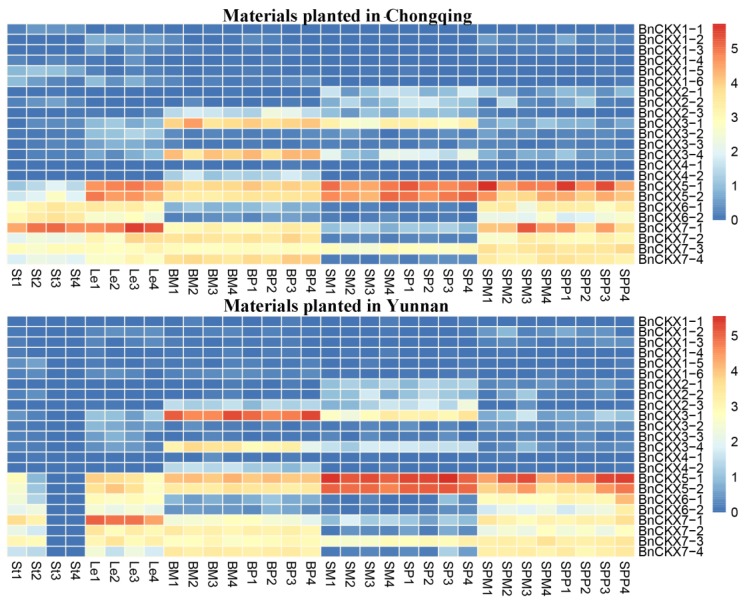
Heatmaps of the expression patterns of the 23 *BnCKX* genes in eight *B. napus* organs in two environments. The expression data were obtained from publically available RNA-sequencing (RNA-seq) data. Le: mature leaves; St: stems; BP: buds on the primary branch; BM: buds on the main stem of the inflorescence; SM and SP: seeds on the main stem of the inflorescence and primary branch, respectively; SPM and SPP: silique pericarps on the main stem of the inflorescence and primary branch, respectively; 1: materials with high yields and low harvest indices; 2: materials with low yields and high harvest indices; 3: materials with high yields and high harvest indices; 4: materials with low yields and low harvest indices.

As shown in the heatmaps (Figure 5), *BnCKX1*, *BnCKX2*, *BnCKX4* exhibited almost no expression in any tissue. Other *BnCKX* family members were expressed at high or low levels in different tissues. For example, *BnCKX5-1* and *5-2* were highly expressed in seeds and silique pericarps, while *BnCKX6-1* and *6-2* were mainly expressed in leaves, stems and silique pericarps but were not expressed in seeds or buds. *BnCKX7-1* was highly expressed in stems and leaves. Members in the same subfamily also showed diverse expression patterns. For example, *BnCKX3-2* and *3-3* were not expressed in any tissues, while *BnCKX3-1* and *3-4* were expressed specifically in buds and seeds. The expression patterns of *BnCKX7-2* and *7-4* were very similar; neither was expressed in seeds or stems. By contrast, *BnCKX7-3* was expressed in all tissues examined at similar levels. In addition, eight DEGs were found in plants with different yields or harvest indices. Notably, the expression levels of *BnCKX5-1*, *6-1*, *6-2*, and *7-1* in siliques were significantly different in the siliques of plants with different harvest indices.

### 3.6. qRT-PCR Measurement of *BnCKXs* Gene Expression in Specific Tissues and at Different Developmental Stages

**Figure 6:**
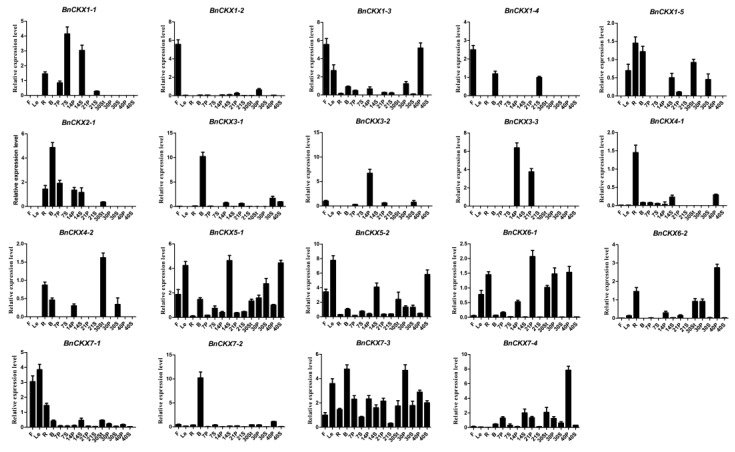
Expression of 19 *BnCKX* genes in different tissues and at different developmental stages. No BnCKX1-6, 2-2, 2-3 and 3-4 expression was detected using quantitative reverse-transcription polymerase chain reaction qRT-PCR. The expression patterns of the 23 *BnCKX* genes in *B. napus* cultivar Zhong Shuang 11 (‘ZS11’) were analyzed by qRT-PCR. F: flowers; Le: leaves; R: roots; B: buds; 30St: stems on day 30; 7S, 14S, 21S, 30S, 40S: seeds collected at 7, 14, 21, 30 and 40 days after flowering, respectively. 7P, 14P, 21P, 30P, 40P: silique pericarps collected at 7, 14, 21, 30, and 40 days after flowering, respectively.

To better understand the characteristics of *BnCKX* gene expression, we analyzed the expression levels of the 23 *BnCKX* genes in roots, stems, leaves, buds, flowers, seeds, and silique pericarps at different developmental stages (7S, 7P, 14S, 14P, 21S, 21P, 30S, 30P) by qRT-PCR. The *BnCKX* genes had diverse expression patterns (Figure 6). Whereas *BnCKX1-6*, *2-2*, *2-3* and *3-4* were not expressed in any tissue examined, the 19 other *BnCKX* genes exhibited tissue-specific, developmental stage-specific, or ubiquitous expression. For example, *BnCKX5-1*, *BnCKX5-2*, and *BnCKX7-3* were expressed in all tissues at different levels, while the other genes were only expressed in specific tissues or at specific developmental stages. For instance, *BnCKX5-1*, *5-2*, *7-1*, and *7-3* were highly expressed in leaves. *BnCKX1-2*, *1-3*, and *1-4* were highly expressed in flowers. However, the expression patterns of some genes of the same subfamily were inconsistent. For example, *BnCKX3-2* and *3-3* were mainly expressed in the silique pericarp, while *BnCKX3-1* was strongly expressed in buds. In addition, both *BnCKX5-1* and *BnCKX5-2* were strongly expressed in 14S and 40S stages but weakly expressed in 21S and 30S. *BnCKX6-1* and *6-2* were mainly expressed in 21P, 30P and 40P but were expressed at low levels in 7P and 14P. Finally, *BnCKX3-3*, *7-3* and *7-4* were highly expressed in 14P, 30P and 40P, respectively. These finding suggest that BnCKX3-3, 5-1, 5-2, 6-1, 6-2, 7-3, and 7-4 might play important roles in the temporal and spatial control of pod development.

### 3.7. Expression of Selected *BnCKX* Genes in Different Plant Materials and in Response to IAA and 6-BA Treatment

**Figure 7:**
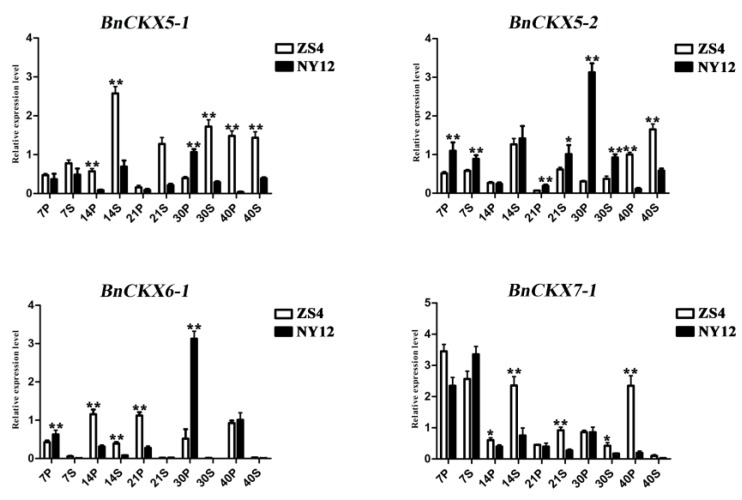
Differentially expressed genes between plant materials with extremely long vs short siliques; the average silique length is 9.80 cm in *B. napus* cultivar Zhong Shuang 4 (‘ZS4’) and 3.50 cm in Ning You 12 (‘NY12’).

Based on the RNA-Seq and ZS11 qRT-PCR data, we selected four genes (*BnCKX5-1*, *5-2*, *6-1*, and *7-1*) that might function in silique development and analyzed their expression patterns in ‘ZS4’ and ‘NY12’ (the average silique length can be seen in Table S1) via qRT-PCR. Variance analysis revealed that the expression levels of these four genes showed significant differences or extremely significant (*p* value < 0.05 or 0.01) between material ‘ZS4’ and ‘NY12’ (Figure 7 and Table S4), suggesting that *BnCKX5-1*, *5-2*, *6-1*, and *7-1* might help determine the length and development of siliques. Notably, *BnCKX5-1* and *7-1* were more highly expressed in 14S, 21S, 30S in ’ZS4’, whereas *BnCKX5-2* was more highly expressed in 7S, 21S, 30S in ’NY12’. Moreover, *BnCKX6-1* was mainly expressed in the silique pericarp, with extremely significant differences in expression between these two materials. *BnCKX6-1* was strongly expressed in 14P and 21P in ’ZS4’ but in 7P and 30P in ’NY12’. Perhaps lower expression levels of *BnCKX6-1* during the early stages of silique development and higher expression levels in the middle-stage of this process lead to greater final silique lengths.

**Figure 8:**
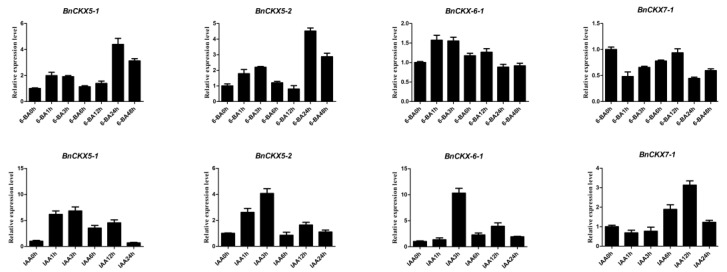
Responses of differentially expressed *BnCKX* genes to 6-benzylaminopurine (6-BA) and indole-3-acetic acid (IAA) treatment. IAA 0 h: control sample; IAA 1, 3, 6, 12, 24 h: sample treated for 1, 3, 6, 12 and 24 hours with IAA; 6-BA 0 h: control sample; 6-BA 1, 3, 6, 12, 24, 48 h: sample treated for 1, 3, 6, 12, 24, and 48 hours with 6-BA. **: *p* <0.01. *: *p* <0.05.

To gain more insight into the roles of *B. napus CKX* genes in stress responses, we used qRT-PCR to examine the expression of four DEGs in response to treatment with the synthetic cytokinin 6-BA or the auxin IAA (Figure 8). *BnCKX5-1, 5-2,* and *6-1* were moderately induced by 6-BA. The expression patterns of *BnCKX5-1* and *5-2* were similar, both were slightly induced at the beginning of 6-BA treatment and reached a peak at 3 h, followed by a decrease, and another peak at 24 h. Perhaps this phenomenon is related to the complex dynamic balance that shifts after application of the exogenous hormone. By contrast, *BnCKX7-1* was downregulated under this treatment. Under IAA treatment, *BnCKX5-1* and *5-2* were significantly induced at 3 h, while *BnCKX7-1* was slightly downregulated, followed by upregulation, reaching a peak at 12 h, and was again downregulated at 24 h. Compared with 6-BA treatment, IAA more strongly induced *BnCKX6-1* expression, with a >10-fold increase in expression at 3 h.

## 4. Discussion

The *B. napus* (AACC, 2n=38) genome comprises the *B. rapa* and *B. oleracea* genomes, which descended from a common ancestor [61,62]. Genome-wide analysis and expression profiling of the *CKX* gene family have been performed in *Arabidopsis* and the diploid *B. rapa*, revealing 7 and 12 *CKX* genes in *Arabidopsis* and *B. rapa*, respectively [5,27]. By contrast, we identified only 23 *BnCKXs* from the tetraploid *B. napus* in the current study, and it contains only 2-6 copies homologs of each *AtCKX* genes due to gene loss and genome shrinkage. By comparing the number of *BnCKX* and *BoCKX* genes identified in each subfamily in the present work with those identified by Liu et al in *B. rapa*, we found that the number of *BrCKX* and *BoCKX* genes identified in each subfamily is equal and that the number of *BnCKXs* is about the sum of *BrCKXs* and *BoCKXs*. In this study, only 3 *BnCKX2* genes were identified, whereas *B. rapa* contains 2 *BrCKXs* and *B. oleracea* contains 2 *BoCKXs*. These results suggested that there may be gene elimination in *BnCKX2s*. By constructing a phylogenetic tree and predicting the gene structures, protein profiles, and conserved motifs of the *BnCKXs*, we found that genes belonging to the same subfamily exhibit similar features. The structures and conserved motifs of these genes might be closely related to their expression patterns. For example, *BnCKX7-1*, *7-2*, and *7-4* share similar gene structures and conserved motifs, as well as expression levels and tissue-specific expression patterns. However, the gene structure and conserved motifs of *BnCKX7-3* are quite different from those of the other CKX members, as is its expression pattern, pointing to a functional division in this gene family.

To better understand the functions and interactions of the 23 BnCKX proteins, we predicted their subcellular localizations. The BnCKX proteins are localized in different cellular compartments, including the chloroplast, cytosol, mitochondria, vacuole, and nucleus. In addition, BnCKX3 proteins contain vacuole-targeting peptides, and AtCKX3-GFP is localized to the vacuole [5]. By contrast, BnCKX6 and BnCKX1-2, -4, -5 and -6 are localized to the mitochondria, indicating that BnCKX6 likely interacts with BnCKX1 in the mitochondria. 

Analysis of *cis*-elements in the promoters of the *BnCKXs* suggested that they play a role in regulating biotic and abiotic stress responses, as well as plant growth. Indeed, *CKX1* expression is induced by cytokinins, abscisic acid, and abiotic stress in maize [63], and *CKXs* are thought to function in the crosstalk between gibberellic acid and cytokinin signaling, thereby regulating plant growth and development [64,65]. In the current study, we predicted that six types of hormone-responsive *cis*-acting elements are present in the promoters of *BnCKX* genes, including auxin-responsive, jasmonate-responsive, gibberellin-responsive, ethylene-responsive, and salicylic acid-responsive elements. However, we did not detect cytokinin-responsive elements, perhaps because only a few such elements have thus far been identified in plants. CKX-overexpressing plants exhibit increased drought and salinity tolerance, as well as enhanced heat stress tolerance [66,67]. In the current study, we identified MBS elements in all *BnCKX* genes, as well as HSE and TCA-elements in 15 and 10 *BnCKX* genes, respectively. These results suggest that most *BnCKX* genes are associated with plant growth and stress responses.

Based on the RNA-Seq data (Table S5), we constructed two heat maps, revealing the diverse gene expression patterns of the *BnCKXs*. In general, the expression patterns of *BnCKXs* in two environments were highly similar, with expression of only half of the 23 *BnCKX* genes detected. However, the expression patterns of members of the same subfamily sometimes differed, such as for the *BnCKX3* and *BnCKX7* subfamilies, suggesting some functional diversification among gene family members. In addition, most *BnCKX* genes were highly expressed in reproductive organs such as buds, flowers or siliques. In *Arabidopsis*, *ckx3* and *ckx5* double mutants form large inflorescences and floral meristems and contain supernumerary ovules, thus leading to an increase in seed set per silique [68]. There were also some discrepancies between the expression patterns of RNA-seq data and quantitative polymerase chain reaction (qPCR) analysis, especially the expression level of *BnCKX1-2, 1-3, 2-4* and *3-4*. One possible reason is that the total RNA for RNA-Seq was pooled from four types *B.napus* lines with extremely high and low harvest index, while the samples for qRT-PCR analysis were collected from ‘ZS11’. On the other hand, the seeds and silique pericarps for RNA-Seq were harvested at 20 days after flowering, and the mature leaves, buds and stems were harvested at flowering stage 63–65. These discrepancies also indicated the complex expression profiles of *BnCKX* genes in different materials and plant development stages. In the current study, we found that the expression level of *BnCKX5* markedly differed not only at different stages of pod development but also in different plant materials. Thus, we hypothesize that BnCKX5 might play an essential role in modulating cytokinin levels, thus regulating silique development. Notably, *BnCKX7-1* was downregulated following exogenous 6-BA treatment, and *Arabidopsis* overexpressing *AtCKX7* showed increased CKX enzymatic activity, ultimately leading to cytokinin deficiency [69]. Based on these findings, we hypothesize that cytokinin deficiency is the ultimate result of *CKX7-1* expression and that the process of cytokinin degradation by CKX enzymes is a dynamic and complex process.

## Figures and Tables

**Table 1:** Complete list of the 23 *BnCKX* genes identified in this study.

Gene Name	Locus Name	Chr.	Location	ORF Length (bp)	Protein			Subcellular Location
					Length (aa)	Mw (Da)	pI	
*BnCKX1-1*	*BnaA04g23980D*	A04	17680020…17682356	1611	536	60,681.24	8.58	chlo: 6, extr: 3, vacu: 2, nucl: 1, plas: 1
*BnCKX1-2*	*BnaA05g02240D*	A05	1254144...1259378	1602	533	60,411.94	8.72	mito: 7, chlo_mito: 6.33333, chlo: 4.5, cyto_mito: 4.33333
*BnCKX1-3*	*BnaC04g01930D*	C04	1500877…1503073	1791	596	68,227.7	8.53	nucl: 4, vacu: 4, chlo: 3, plas: 1, extr: 1
*BnCKX1-4*	*BnaC04g47740D*	C04	46681907…46684463	1641	546	61,665.39	9.2	mito: 7.5, cyto_mito: 4.5, chlo: 3, nucl: 2
*BnCKX1-5*	*BnaA03g19500D*	A03	9235344...9237387	1638	545	61,439.11	8.72	mito:4,chlo: 4, cyto: 3, nucl: 2
*BnCKX1-6*	*BnaC03g23380D*	C03	13022610...13024649	1599	532	60,320.83	8.8	mito: 5, chlo: 4, nucl: 2, cyto: 2
*BnCKX2-1*	*BnaA07g35940D*	A07_random	5099…13485	2307	768	87,023.55	5.86	nucl: 7, cyto: 4, chlo: 2
*BnCKX2-2*	*BnaC07g01710D*	C07	2422452...2425861	1011	336	38,171.83	5.83	chlo: 4, cyto: 2, extr: 2, vacu: 2, mito: 1.5, mito_plas: 1.5, nucl: 1
*BnCKX2-3*	*BnaA09g52930D*	A09_random	753807...758243	1455	484	53,519.55	5.96	vacu: 4, chlo: 3, extr: 3, plas: 2, cyto: 1
*BnCKX3-1*	*BnaA02g08420D*	A02	4,079,790…4,082,972	1554	517	58,722.84	5.7	vacu: 6, mito: 2, extr: 2, golg: 2, plas: 1
*BnCKX3-2*	*BnaA10g28940D*	A10_random	1464876…1467891	1437	478	54,173.56	5.93	vacu: 5, extr: 3, mito: 2, plas: 2, chlo: 1
*BnCKX3-3*	*BnaC09g33450D*	C09	36760027…36762978	1437	478	54,183.47	5.78	vacu: 7, extr: 4, cyto: 1, mito: 1
*BnCKX3-4*	*BnaCnng41060D*	Cnn_random	39653407…39656550	1557	518	58,855.91	5.83	vacu: 6, extr: 3, golg: 2, mito: 1, plas: 1
*BnCKX4-1*	*BnaA03g49660D*	A03	25641300…25644280	1551	516	57,072.39	5.51	chlo: 5, cyto: 2, mito: 2, vacu: 2, E.R.: 2
*BnCKX4-2*	*BnaC07g50850D*	C07_random	2732541…2736266	1551	516	57,301.75	5.62	vacu: 4, chlo: 2, mito: 2, extr: 2, golg: 2, cyto: 1
*BnCKX5-1*	*BnaAnng09190D*	Ann_random	9695112...9698437	1596	531	59,613.56	5.62	extr: 5, chlo: 3, vacu: 3, cyto: 1, mito: 1
*BnCKX5-2*	*BnaC06g36220D*	C06	34801906…34804721	1608	535	60,144.10	5.53	vacu: 8, extr: 3, cyto: 1, mito: 1
*BnCKX6-1*	*BnaA09g40400D*	A09	28427957...28430033	1350	449	50,468.31	7.3	mito: 7, nucl: 4, chlo: 2
*BnCKX6-2*	*BnaC08g32840D*	C08	31616324...31618400	1602	533	60,109.91	8.55	mito: 7, chlo: 1, nucl: 1, cyto: 1, plas: 1, extr: 1, E.R.: 1
*BnCKX7-1*	*BnaA02g05340D*	A02	2433263…2436786	1554	517	57,559.29	5.2	cyto: 6, nucl: 3, cysk: 2, chlo: 1, plas: 1
*BnCKX7-2*	*BnaA10g14370D*	A10	11435086…11438436	1578	525	57,864.41	4.92	cyto: 11, chlo: 1, plas: 1
*BnCKX7-3*	*BnaA10g24380D*	A10	15919508...15923282	1704	567	61,909.23	7.93	chlo: 7.5, chlo_mito: 7, mito: 5.5
*BnCKX7-4*	*BnaC09g36710D*	C09	40060399…40063686	1395	464	51,901.91	5.45	chlo: 8, cyto: 4, nucl: 1

## Supplementary Materials

The following are available online at http://www.mdpi.com/2073-4425/9/3/168/s1, Table S1: Silique length of extremely materials at several stages of pod development, Table S2: Primers used for quantitative reverse-transcription polymerase chain reaction (qRT-PCR) analysis of *BnCKX* genes, Table S3: Stress- and hormone-responsive elements in the promoter regions of *BnCKX* genes, Table S4: The expression levels differences of selected four genes by variance analysis, Table S5: RNA-Seq data of 23 *BnCKX* genes.
